# Wildlife as Source of Human *Escherichia coli* O157 Infection

**DOI:** 10.3201/eid2312.171210

**Published:** 2017-12

**Authors:** Brian Crook, Helena Senior

**Affiliations:** Health and Safety Laboratory, Health and Safety Executive, Buxton, United Kingdom

**Keywords:** *Escherichia coli*, environmental, infection risk, wildlife, zoonoses, England, rabbits, bacteria, Shiga toxin, STEC, Shiga toxin–producing *Escherichia coli*

**To the Editor:** The article by Probert et al. (*1*) highlighted that wild animals (in this case, deer) can act as a reservoir of Shiga toxin–producing *Escherichia coli* (STEC) O157 infection. Our previous research (*2*) broadens this to include wild animals as STEC O157 carriers. In our study, an outbreak of STEC O157 infection in eastern England was epidemiologically linked to visiting a wildlife park. Unlike in petting zoos, the visitors had no direct contact with the animals. Transmission of infection was attributed to contact with the feces of wild rabbits (*Oryctolagus cuniculus*) in a play area; the rabbits had been in contact with STEC O157–positive cattle in an adjacent field (*3*). To prove this hypothesis, we identified rabbit populations living on farms with STEC O157–positive cattle. We trapped the rabbits humanely in cages, collected their feces during their confinement before release, and tested the feces for STEC O157 by culture and PCR. Of 97 samples collected in the summer, 8 (8.2%) were positive by culture; these samples came from 4 of 6 farms in the study. By PCR analysis, 20 of 97 (20.6%) samples were positive. None was positive during the winter, when cattle were housed indoors, suggesting a link between STEC O157 positivity in cattle and rabbits. In conclusion, when outbreaks of this serious human infection are linked to the rural environment, it is necessary to take wildlife into consideration, both as a reservoir of transmission and as carriers. Equally, persons should not underestimate the necessity of good hand hygiene if they have contact with wild animal feces.
